# Mixed Infection of *Mycobacterium szulgai*, *M. lentiflavum*, and Gram-Negative Bacteria as a Cause of Death in a Brown Caiman *Caiman crocodylus*: A Case Report

**DOI:** 10.3390/vetsci9030133

**Published:** 2022-03-13

**Authors:** Aleksandra Maluta, Magdalena Zając, Monika Krajewska-Wędzina, Dariusz Wasyl, Kim Heckers, Anna Didkowska, Krzysztof Anusz

**Affiliations:** 1 Veterinary Clinic and Hospital for Exotic Pets OAZA, ul. Potocka 4, 01-652 Warsaw, Poland; 2 Department of Microbiology, National Veterinary Research Institute, Al. Partyzantów 57, 24-100 Puławy, Poland; magdalena.zajac@piwet.pulawy.pl (M.Z.); monika.krajewska@piwet.pulawy.pl (M.K.-W.); wasyl@piwet.pulawy.pl (D.W.); 3 Laboklin Labor fur Klinische Diagnostic GmbH& C.O. KG, 97688 Bad Kissingen, Germany; heckers@laboklin.com; 4 Department of Food Hygiene and Public Health Protection, Institute of Veterinary Medicine, Warsaw University of Life Sciences (S.G.G.W.), Nowoursynowska 166, 02-787 Warsaw, Poland; anna_didkowska@sggw.edu.pl (A.D.); krzysztof_anusz@sggw.edu.pl (K.A.)

**Keywords:** caiman, mycobacteriosis, *Mycobacterium lentiflavum*, *Mycobacterium szulgai*, nontuberculosis, reptiles, *Salmonella*

## Abstract

This paper describes a fatal case of nontuberculosis mycobacteriosis in a four-year-old brown caiman kept in captivity. Although the clinical signs were asymptomatic, severe gross lesions were observed, namely necrotic inflammation of the intestines and granulomatous hepatitis. Microbiological and histopathological examination performed on the tissues collected postmortem revealed a mixed infection of *Mycobacterium lentiflavum* and *Mycobacterium szulgai*, secondarily mimicked with *Salmonella Coeln*, *Aeromonas hydrofila*, *Citrobacter freundii*, and *Providencia rettgeri*. Those microorganisms are not only potentially pathogenic to reptiles, but also have a zoonotic importance for humans. Our findings clearly demonstrate the importance of educating owners and maintaining hygiene rules when handling reptiles.

## 1. Introduction

The caimans (*Caimaninae*) belong to the reptile family, which includes species from the genera *Caiman*, *Melanosuchus*, and *Paleosuchus*. Nowadays, caimans, like other exotic animals, are increasingly being bought as pets. These purchases raise not only ethical questions but also those concerning public health. As there is no exact knowledge about the threat posed by human contact with these exotic animals, each described case of infection in such animals kept as pets, may provide valuable information. Owners should be aware that close contact with their pets can represent a source of zoonotic infection. One of the groups of potentially zoonotic bacteria are nontuberculous mycobacteria (NTM), which are emerging as pathogens in both humans [1,2] and animals [3]. 

In reptiles, mycobacteriosis usually manifests as granulomatous lesions in affected organs or tissues [4]. Clinical signs may differ depending on the system involved; however, they are usually nonspecific [5]. At necropsy, white-grayish nodules are observed, and histopathologic examination reveals typical granulomatous inflammations with multinucleated giant cells. However, unlike mammalian tubercles, reptilian tubercules do not demonstrate any calcification [5]. Mycobacteria infections have been reported in lizards [6], snakes [7], turtles [8], and in crocodiles [4,9]. The mycobacteria were identified as *Mycobacterium* (*M.*) *chelonae*, *M. fortuitum*, *M. intracellulare*, *M. marinum*, *M. phlei*, *M. smegmatis*, *M. ulcerans*, *M. confluentis*, *M. haemophilum*, *M. hiberniae*, *M. neoaurum*, and *M. nonchromogenicum* [5].

Various Gram-negative bacteria have also been isolated from clinical cases in crocodiles; however, their pathogenicity is not clear. For example, although *Salmonella* spp. is considered a physiological component of healthy reptilian gut flora, the animals might, in some circumstances, develop clinical signs, usually as a secondary causative agent [10]. *Salmonella* spp. is considered to be a potential threat for owners of all pets, including reptiles [11,12,13]. Most studies indicate that the key public health problems associated with crocodiles and alligators arise due to consumption of their meat, such as *Salmonella* spp. infection [14], parasites [15], heavy metal poisoning [16], or allergies [17]. In this study, we wanted to highlight the possible risks for those keeping the animals as pets. Although the most widely-known threat in this regard is salmonellosis, the present study also examined the zoonotic potential of atypical mycobacteria.

This report describes the first case of mycobacteriosis recorded in a brown caiman (*Caiman crocodylus*) caused by a mixed infection of *M. lentiflavum* and *M. szulgai*, and a simultaneous co-infection with a number of Gram-negative bacterial species. 

## 2. Materials and Methods

### 2.1. Animal Description

A 2.30 kg, four-year-old female brown caiman was placed on an display in a pet shop. The caiman, purchased from an exotic animal wholesaler in Germany a few months previously, was housed alone in a 2.5 m long terrarium, comprising part land and part water. Water temperature was maintained at 24 °C and air was 28 °C. No filtration system was used and the water was changed once a month. The reptile was fed once a week with freshwater fish, mice, or rats. Four months after purchase, the caiman refused food for two consecutive weeks, but no other abnormalities had been observed. The animal died two weeks after the second episode of food refusal.

### 2.2. Postmortem Examination

A routine postmortem examination was carried out on the caiman. The procedure included a description of the animal, an external and internal examination, and a detailed inspection of individual organs. A protocol was drawn up during the examination. The postmortem examination was carried out in accordance with the principles of biosecurity. During the examination, organ samples (liver and small intestine) were collected for histopathological and microbiological examination.

### 2.3. Histological and Microbiological Examination

Liver and small intestine samples were fixed in a 10% neutral buffered formalin and sent to a commercial laboratory for histopathological examination. The remainder of the affected tissues were subjected to bacteriological examination. Two tissue liver and intestine samples with a size of 1.5 × 1.0 × 0.3 cm and 2.2 × 1.2 × 0.9 cm were tested. H&E (hematoxylin and eosin), PAS (periodic acid–Schiff) and Ziehl–Neelsen staining were performed according to standard operation procedures. 

The liver tissue samples were homogenized in 0.85% NaCl (saline fluid) and split into two portions for bacterial culture, including mycobacteria. One part of the liver sample was decontaminated in 5% oxalic acid and flushed twice with a 0.85% NaCl, in accordance with the guidelines of the central veterinary office [18]. The sediments were inoculated onto four Stonenbrink (S) and four Petragnani (P) solid media. All reagents used in this stage were prepared by the media department of the National Veterinary Research Institute (Pulawy, Poland). To accommodate the different growth requirements of mycobacteria, half of the cultures were incubated at 25 °C (+/−2 °C), and the remaining, at 37°C (+/−2 °C) for four weeks, with weekly readings. The mycobacteria isolates were identified based on their growth on S and P slants and on their colony morphology. 

Part of the sediment was suspended in PANTA^®^ reagent to inhibit the growth of other microorganisms, placed in a Middlebrook^®^ liquid medium tube, and introduced to the BD BACTEC MGIT 960 mycobacterial liquid culture system (Becton, Dickinson and Company, New York, NY, USA). The BD BACTEC MGIT 960 is a fully automated system used to detect the growth of mycobacteria in culture based on the fluorescence of a dye blocked by oxygen, which is metabolized by the bacteria [19]. All reagents used in this step were manufactured by Becton, Dickinson and Company (Franklin Lakes, NJ, USA). 

The final identification was performed with the GenoType Mycobacterium CM assay (Hain Lifescience GmbH, Nehren, Germany). In addition, DNA isolation and amplification were performed using the Genomic Mini AX Bacteria kit (A&A Biotechnology, Gdynia, Poland) in accordance with the manufacturer’s instructions (https://www.aabiot.com/en/download?code=20b7767a5e0776259eb402975db675134e05efaa, accessed on 10 March 2022). 

A second subsample (nontreated) was streaked directly onto a blood agar (homemade) and incubated at 37 °C. Colonies with different morphology were streaked on a nutrient agar (Oxoid, Hampshire, United Kingdom) and identified on ID32E (Biomerieux, Marcy l’Etoile, France) and MALDI-TOF (Bruker, Germany). *Salmonella* isolates were serotyped according to White-Kauffmann-Le Minor [20].

## 3. Results

### 3.1. Postmortem Examination

**Figure 1:**
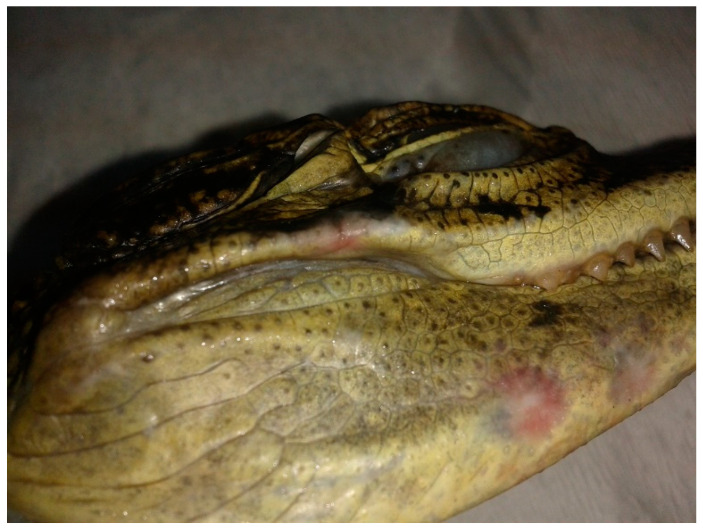
Round pale lesions on the skin of the mandible of the brown caiman.

**Figure 2:**
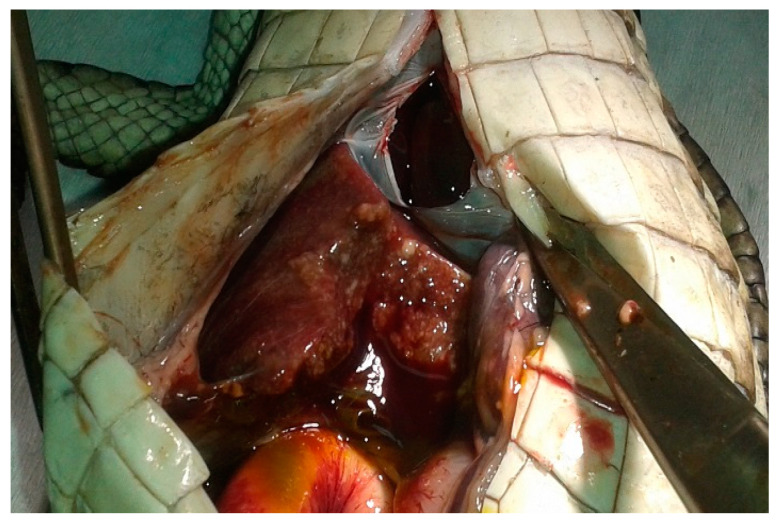
Enlarged liver with white to yellowish nodules.

Postmortem examination revealed round, pale lesions on the skin of the mandible (Figure 1). At necropsy, the liver was enlarged and covered with multiple, discrete, scattered, white to yellowish, 1–3 mm diameter nodules (Figure 2). Similar nodules were observed in the lungs. The stomach was empty and the intestinal lumen was reduced. The wall of the small intestine was thickened with a white to yellowish coating. No gross lesions were visible in any other organs or tissues.

### 3.2. Histological and Microbiological Examination

**Figure 3:**
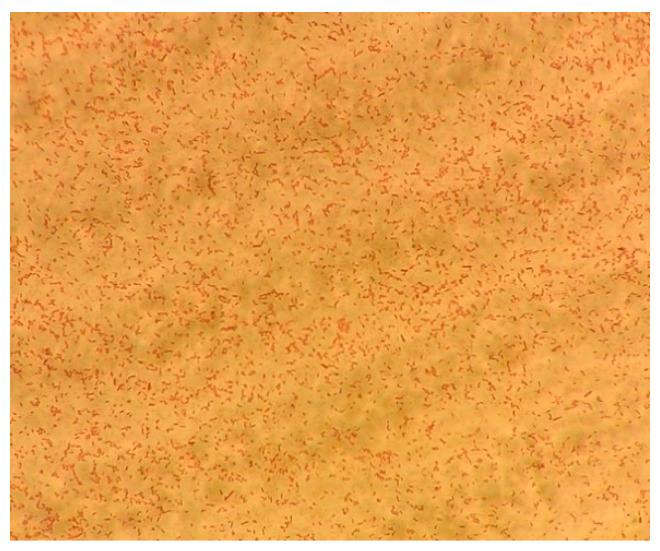
Intralesional acid-fast rod-shaped bacteria with Ziehl–Neelsen staining.

Histological examination revealed the presence of a thick intestine wall composed of fibroblastic material infiltrated with erythrocytes, remnants of leucocytes, and necrotic foci. The tissue was diffusely infiltrated by rod-shaped bacteria. In the liver sample, a severe diffuse granuloma was found; this area was inflamed, and numerous intralesional acid-fast rod-shaped bacteria were present (Figure 3). No fungal structure was observed. 

*Mycobacterium lentiflavum* and *Mycobacterium szulgai* strains were cultured from the liver tissue. Colonies visible on the solid media (S and P) were smooth with typical moist characteristics. The isolates on the P medium were more glistening and yellow. 

These were accompanied by a number of other bacteria: *Aeromonas hydrofila*, *Citrobacter freundii*, *Providencia rettgeri*, and *Salmonella* Coeln.

## 4. Discussion

Many opportunistic bacteria can be responsible for disease in reptiles, and most elicit unspecific symptoms which do not allow for timely diagnosis and efficient treatment. In the described case, only negligible symptoms were observed prior to the animal’s death; however, severe lesions were noted upon postmortem examination, including necrotizing inflammation of the intestines and diffuse granuloma-forming hepatitis due to mycobacteriosis caused by two nontuberculous mycobacteria: *Mycobacterium szulgai* and *M. lentiflavum*.

*Mycobacterium szulgai* has previously been described as a cause of granulomatous pneumonia in a captive freshwater crocodile [8] and systemic infection in a brown caiman [21]. *M. lentiflavum* was described as NTM in 1996 [22], and is known to be responsible for chronic pulmonary diseases in humans, particularly among immunocompromised, HIV-positive patients [23,24]. In addition, in a study of 396 samples of water, aquatic plants and sediments collected from 13 water-related facilities in the Czech Republic, *M. lentiflavum* was also isolated from the drinking water reservoir; this was the first isolation of potentially pathogenic mycobacteria from a freshwater environment [24]. 

The novelty of the current report is its simultaneous detection of two mycobacteria species able to give rise to the observed clinical picture. It is impossible to conclude which was the primary cause or if both infections occurred in parallel from common or separate sources. The clinical picture could also have been obscured by other bacteria which were a part of the natural microflora of reptiles [25,26,27,28,29]. Such bacteria have also been reported from clinical lesions in other animals [29,30,31].

Noteworthy, some of the currently identified bacteria are potentially zoonotic for humans. Many epidemiologic studies suggest that the water environment is the principal source of human exposure to NTM [24,32,33]. Reptiles and fish are often household pets, and if colonized, can be a source of pathogens for owners or other people exposed directly or indirectly to the animal via a contaminated environment [32,34,35]. Indeed, the incidence of mycobacterial diseases, particularly pulmonary mycobacteriosis, is growing in Europe and on other continents [35,36]. For this reason, animal keepers should be made aware of the potential dangers associated with their pets, and the importance of maintaining hygiene and visiting a veterinarian in the event of any disturbing clinical signs. Reptiles should be kept out of rooms where food is prepared and consumed. These areas should not be used to clean reptile habitats; ideally, they should be cleaned outside the home. If this is not possible, the area should be thoroughly disinfected after cleaning. 

Treatment is complicated by the problems associated with selecting appropriate microbiological methods for differential diagnosis and the resistance of mycobacteria to many antimycobacterial drugs [37]. It should be remembered that the treatment of mycobacteriosis, both in humans and animals, requires experience and cooperation between microbiologist and clinician. Moreover, in Europe at least, reptiles intended as pets are often bred abroad and may carry exotic pathogens with them when imported [34,35,38,39]. *Salmonella* is a well-known component of reptile microflora [40,41,42], and many cases of reptile-associated salmonellosis (RAS) have been reported, especially in children under five years old [43,44]. As their immune system is not fully developed, and their greater likelihood to put their fingers in their mouth, children under five years old are not recommended to have reptiles as pets. The same rule applies to the elderly or those with a reduced immune status [45]. Two cases of *Salmonella* with zoonotic potential have also been noted specifically in captive caimans—*S.* Infantis and *S.* Nottingham. Our isolation of the serovar *S. Coeln* is the first report of its presence in reptiles in Poland; despite being rarely observed, it is nevertheless listed among the 20 most frequent serovars causing human salmonellosis in Europe [46]. Although preventive actions, such as monitoring for *Salmonella* spp., are recommended in caiman ranching facilities [47], our findings demonstrate that such measures should be also considered in animals kept as pets. 

Furthermore, it should be remembered that reptiles also serve as reservoirs for other pathogens which can cause illness in humans. In caimans specifically, cases of zoonotic bacteria, such as *Leptospira* spp. [48] and *Arcobacter* spp. [49] have been confirmed, and a number of parasites have been recorded [50]; such infection is also a potential cause for concern by pet owners. Another potential threat to caiman owners is the possibility of a caiman bite [51]. Although data on the microbiology of wounds in humans caused by crocodiles and alligators are limited, a wide range of bacteria have been associated with the oral environment of crocodilians; these have been cultured as *Citrobacter* spp., *Aeromonas hydrophila*, *Clostridium* species, *Entercoccus* species, *Enterobacter agglomerans*, *Pseudomonas aeruginosa*, and *Burkholderia psudomallei*, among others [52]. 

However, as long as proper hygiene is maintained, pet reptiles do not pose a significant health risk to their owners. Strict attention to cage hygiene, regular sanitation, and personal hygiene after handling the individual animals are always advised to minimize the exposure to zoonotic pathogens and their spread. In this regard, veterinarians have an important role in informing reptile owners about possible hazards and hygiene standards.

## 5. Conclusions

This is the first report of the simultaneous detection of mycobacteria species in a caiman, which was able to develop the observed clinical picture. Importantly, our findings emphasize that all isolated bacteria are potentially zoonotic, and as such, there is a need to raise awareness among owners to maintain hygiene rules when handling reptiles.

## Data Availability

The raw data are available at the corresponding author.
